# Ethnopharmacological survey of herbal remedies used for treatment of various types of cancer and their methods of preparations in the West Bank-Palestine

**DOI:** 10.1186/s12906-016-1070-8

**Published:** 2016-03-08

**Authors:** Nidal Amin Jaradat, Rowa Al-Ramahi, Abdel Naser Zaid, Ola Ibrahim Ayesh, Ahmad Mustafa Eid

**Affiliations:** Department of Pharmacy, Faculty of Medicine and Health Sciences, An-Najah National University, P.O. Box 7, Nablus, Palestine

**Keywords:** Ethnopharmacology, Anticancer, Herbal remedies, Traditional use, *Ephedra alata*

## Abstract

**Background:**

Plants have been the primary source of medicines since life on earth; more than 50 % of existing cancer treatments are derived from plants.

**Methods:**

An ethnopharmacological survey of herbal remedies used in cancer treatment was carried out in the West Bank/ Palestine. A questionnaire was distributed to one hundred and fifty herbalists, traditional healers and rural dwellers. Collected information included the names of plants, the used parts, types of cancers for which these plants were used and also their methods of preparation. To identify the most important species used, Factor of informant’s consensus (F_ic_), Fidelity level (Fl) and the Use-value (UV) were calculated.

**Results:**

Collected data has shown that 72 plants are utilized for treatment of cancer, belonging to 44 families; from them Compositae and Lamiaceae were the most common. Leaves and fruits were the most commonly used parts, while decoctions, infusions and syrups were the main methods of preparation. Lung cancer was the most common type of cancer treated with these plants and *Ephedra alata* was the most commonly used plant for treatment of cancer in Palestine. The F_ic_ was high for all the plants; Fl was 100 % for many plants, the highest UV (0.72) was for *Ephedra alata*.

**Conclusions:**

This study showed that many herbal remedies are still used by herbalists in Palestine for treatment of cancer; some of them have been approved scientifically while others are not. A combined effort between informants and scientific institutions working in this field can help in the discovery of new anticancer agents. Moreover, scientists must explore the most suitable method of extraction, formulation and dose determination in order to achieve the best benefits from these herbals.

## Background

The Holy Land/ Palestine has miscellaneous ethnic groups (Muslims, Christians, Druze, Jews from East and West and Samaritans), so its characteristic cultures are numerous and varied, including folkloric herbal medicine. However, these traditions in herbal remedies have waned over hundreds of years. Palestine is a unique land, in its ecological diversity due to its geographical location between Africa, Asia and Europe. Different zoogeographic, climatic, and phytogeographic zones covered Palestine, creating great biological multi-diversity [1, 2]. In addition to that it was as an important international trade crossroad from ancient times, between North Africa, East Asia and West Europe; this added to its culture in herbal medicines [3–5].

Plants provide a continual source of medicines for animals and humans; they have been used since ancient times in crude forms as decoctions, syrups, liniments, powders, infusions and ointments [6, 7]. Evidence of medicinal plant use around 60 000 years ago was found in a cave discovered in 1960 in the Middle East [8, 9]. In recent time, people in both developed and developing countries utilize herbal medicines for improving their health [10–12]. According to the World Health Organization (WHO) evaluations, about 80 % of populations in developing countries have utilized ethno-medicines for their health care requirements and more than 60 % of cancer patients have used natural plant products with vitamins in fighting this disease [13–15]. While 50 % of modern pharmaceutical medications in clinical practice are derived from plants, many of them have strong anticancer effects [16, 17].

According to the WHO and the American Cancer Society about eight million died from cancer and about fourteen million new cancer cases occurred in 2012. The highest percentages of patients were with lung, breast and colorectal cancers respectively. WHO also estimates a substantive increase up to nineteen million new cases of cancer per year by 2025, due to the growth of global population. More than half of all cancers and cancer deaths occurred in less developed countries, and these proportions may be further increased [18–20].

Nowadays, chemotherapeutic anticancer agents are the most common method of treatment, but they may cause serious side effects and toxicity [21–23].

Due to the high death rate among patients with cancer and the hazardous side effects and adverse reactions of the radiotherapy and chemotherapy, cancer patients often start seeking alternative methods of treatments, like herbal medicine with or instead of conventional medicine [24–26].

## Methods

**Fig. 1:**
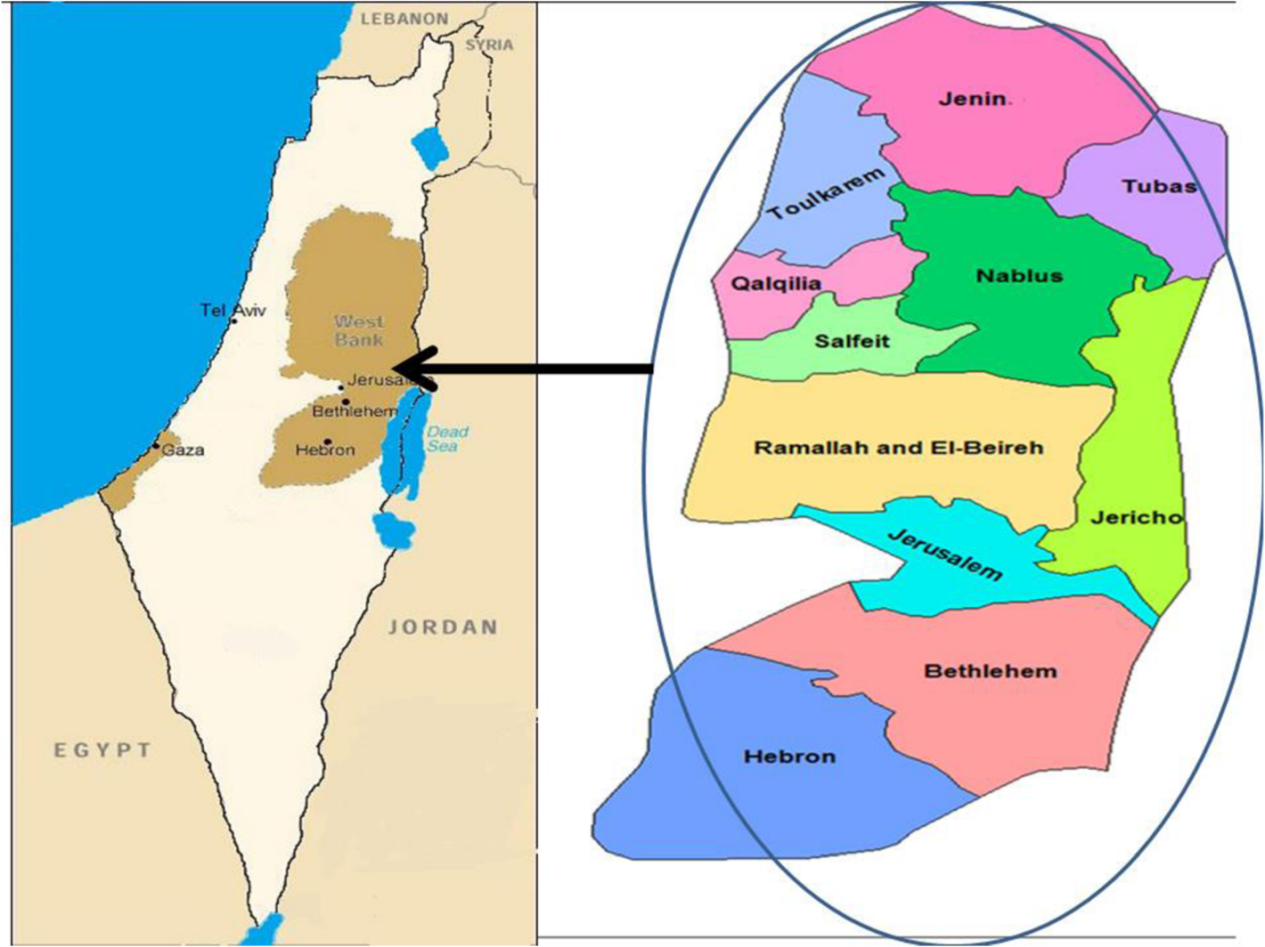
Map of the West Bank/ Palestine showing all surveyed regions

An ethnopharmacological survey on herbal remedies used for treatment of various types of cancer was conducted from March 2015 to June 2015. Areas visited included all regions of the West Bank/Palestine; Nablus, Jenin, Tubas, Toulkarm, Salfeit, Qalqilya, Ramallah, Jericho, Jerusalem, Bethlehem and Hebron (Fig. 1).

The study aims, protocols and the informed consent forms were approved by the Institutional Review Board (IRB) at An-Najah National University (IRB archived number 25/Jan/ 2015). The study was conducted in accordance with the requirements of the declarations of Helsinki.

The study was carried out by interviews with herbalists, traditional healers and rural dwellers that used herbal remedies in the treatment of different types of cancer. The number of registered herbalists in the West Bank is 222. According to the Raosoft calculator the minimum sample should be 141 participants, so we included 150 [27]. These informants represented most of the practitioners in this field in the West Bank (Sociodemographic characteristics are presented in Table 1).

**Table 1:** Sociodemographic characteristics of the respondents

Variable	N (%)
Gender	
Male	128 (85.3)
Female	22 (14.7)
Education level	
Uneducated	26 (17.3)
Elementary	14 (9.3)
Secondary school	28 (18.7)
High secondary school	53 (35.3)
Undergraduate	27 (18)
Graduate (higher education)	2 (1.3)
Residency	
Bethlehem	13 (8.7)
Hebron	13 (8.7)
Jenin	21 (14.0)
Jericho	9 (6.0)
Jerusalem	10 (6.7)
Nablus	20 (13.3)
Qalqilya	7 (4.7)
Ramallah	14 (9.3)
Salfeit	18 (12.0)
Tubas	7 (4.7)
Toulkarm	18 (12.0)
Age (mean ± SD) years	54.7 (14.3)
Experience (mean ± SD) years	34.8 (14.3)

A convenience sample of herbalists and practitioners from various regions were met by researchers and asked to answer a face to face questionnaire. They were interviewed in Arabic after getting their verbal consent only once.

Statistical analyses were performed by using Statistical Package for Social Sciences (SPSSversion17.0). Mean ± standard deviation were computed for continuous data. Frequencies and percentages were calculated for categorical variables. Means were compared using Student’s *t*-test. Categorical variables were compared using Chi-squared and Fisher’s exact tests, as applicable. A *p*-value of less than 0.05 was considered to be statistically significant for all analyses.

Questionnaires were administered through personal contact discussions. This method is an effective and easy option of data collection. This survey aimed to obtain information on the names of plants commonly used in the treatment of cancer, the type of cancer treated by herbs, the methods of preparing and the parts used for administration. Interviews were conducted in the Arabic language of the informants. Names of plants were translated later to English and Latin. In most cases, the interviews often started in the form of informal discussions to gain the confidence of the interviewees.

All of the 72 plant materials were collected from the interviewees (herbalists, traditional healers and rural dwellers) and kept in special glass frames and later identified by the pharmacognosist Dr. Nidal Jaradat. The identity of each plant species mentioned by the interviewees was confirmed and verified by using photographs and live specimens. A medicinal use was accepted as valid only if it was mentioned by at least three independent herbal practitioners. Samples of these collected herbs were given a herbarium specimen number as shown in Table 2 and voucher samples were kept at the Pharmacognosy Laboratory of the Department of Pharmacy at An-Najah National University, Faculty of Medicine and Health Sciences (Table 2).

**Table 2:** Medicinal plants used for treatment of cancer in the West Bank regions/Palestine

	Scientific names/Common names/Arabic names/Voucher specimen code	Family	Part used, method of preparation (herbal formulation) and dosages	Cancer Type	Preparation method and administration	UV
1.	*Allium cepa* L./Onion / Basal/ Pharm-PCT-2703	*Amaryllidaceae*	Bulb/About 20–40 ml of the bulb juice is to be given orally 7–8 times daily for four weeks.	Lung and stomach	Fresh bulb (oral)	0.29
2.	*Allium sativum* L./ Garlic/Thom/ Pharm-PCT-2704	*Amaryllidaceae*	Bulb/2–3 fresh cloves are eaten raw three times daily with meals.	Lung, Esophageal and breast	Fresh bulb (oral)	0.46
3.	*Mangifera indica* L./ Mango/ Manga/ Pharm-PCT-2725	*Anacardiaceae*	Fruits/About 300 ml of fresh fruit juice is to be given orally three times daily.	Colon	Fresh juice (oral)	0.09
4.	*Pistacia palaestina* Boiss./ Mastic tree, Lentisk/ Sirees/ Pharm-PCT-1870	*Anacardiaceae*	Leaves/Powdered leaves mixed with goat fat as paste, applied externally twice daily on cancer area.	Skin	Paste (topical)	0.59
5.	*Annona muricata* L. / Soursop/ Keshta/ Pharm-PCT-2726	*Annonaceae*	Fruit/One fresh fruit boiled with 100 ml syrup for five minutes; 20 ml of the produced syrup is to be given orally three times daily.	Bladder, prostate and colon	Syrup (oral)	0.32
6.	*Daucus guttatus* Sm. / Wild Carrot/ Jazar barry/ Pharm-PCT-832	*Apiaceae*	Seeds/About 100–130 powdered seeds steeped in water for 12 hours; 100 ml from the produced infusion is to be orally given four times daily.	Skin	Infusion (oral)	0.02
7.	*Petroselinum crispum* (Mill.) Fuss/ Parsley/ Bokdonas/ Pharm-PCT-2739	*Apiaceae*	Fruits/About 500–600 grams from the ground dry fruits boiled with one liter water for 30 minutes; 300 ml of this decoction is to be given orally three times daily.	Kidney and bladder	Decoction (oral)	0.16
8.	*Calotropis procera* (Aiton) Dryand./ Apple of Sodom, (mudar)/ A’oshar basek/ Pharm-PCT-472	*Apocynaceae*	Aerial parts (latex)/ Ten grams from air dried latex from the fruits is boiled with 40 ml water; 20 ml of this decoct is to be given twice daily.	Skin	Decoction (oral)	0.61
9.	*Catharanthus roseus* (L.) G.Don/ Vinca/ Wanaky/ Pharm-PCT-2728	*Apocynaceae*	Entire plant/About 25grams from the powdered plant mixed with 30 ml water; this paste is applied topically once daily.	Skin	Paste (topical)	0.47
10.	*Nerium oleander* L./Oleander/ Dafla/ Pharm-PCT-1636	*Apocynaceae*	Entire plant/About 20 grams from the powdered plant are mixed with 50 grams lanolin; this cream is applied topically on the skin cancer area directly 4–5 times daily.	Skin	Cream (topical)	0.53
11.	*Arum dioscoridis* Sm./ Spotted arum/ loof mobarkash/ Pharm-PCT-243	*Araceae*	Leaves/Boil about 10 grams of the dried leaves with 100 ml water, fifty ml of this decoction is to be given orally before meal.	Liver and stomach	Decoction (oral)	0.66
12.	*Arum palaestinum* Boiss./ Cuckoo pint / loof/ Pharm-PCT-246	*Araceae*	Leaves/Boil about 10 grams of the dried leaves with 150 ml water, fifty ml of this decoction is to be given orally three times daily before meals.	Liver, colon, kidney and breast	Decoction (oral)	0.63
13.	*Polygonatum multiflorum* (L.) All./ David's harp / Khatem Soleyman/ Pharm-PCT-2727	*Asparagaceae*	Rhizomes/Steep 2 grams from the powdered rhizomes with 100 ml water for 5 hours; 20 ml from this infusion is to be given orally three times daily.	Liver, brain and spinal cord	Infusion (oral)	0.60
14.	*Brassica oleracea* L./Cabbage/ malfof/ Pharm-PCT-1930	*Brassicaceae*	Leaves/100 ml from the fresh cabbage leaf juice is to be given 7–9 times daily.	Liver	Fresh juice (oral)	0.46
15.	*Sinapis arvensis* L./Wild mustard/ Khardal/ Pharm-PCT-2284	*Brassicaceae*	Seeds/Powdered seeds (2 g) given orally twice daily.	Bone	Powder (oral)	0.47
16.	*Capparis spinosa* L./Caper bush/ Cobar/ Pharm-PCT-496	*Capparaceae*	Roots/Crushed fresh roots (100 grams) mixed with 10 ml water, made into paste then applied topically once daily.	Bones cancer	Paste (topical)	0.47
17.	*Colchicum hierosolymitanum* L./ Colchicum/Lohlah/Pharm-PCT-644	*Colchicaceae*	Seeds/50 grams from the ground seeds mixed with 100 grams lanolin a are applied topically once daily on the tumor area.	Skin	Cream (topical)	0.53
18.	*Achillea aleppica* DC./ Yarrow/ Kaysoom/ Pharm-PCT-16	*Compositae*	Aerial parts/Steep 15grams from the plant with 100 ml water for 2 hours, 10 ml from this infusion is to be given internally twice daily.	Liver	Infusion (oral)	0.11
19.	*Cichorium endivia* L./Common chicory/ Shokar/ Pharm-PCT-617	*Compositae*	Flowers/Steep 100 grams from the plant with 100 ml water for 2 hours; 30 ml from this infusion is to be given once daily.	Stomach and colon	Infusion(oral)	0.22
20.	*Inula viscosa* (L.) Aiton/False Yellow head/ Tayon/ Pharm-PCT-2738	*Compositae*	Leaves/ Boil about 20 grams from the flowers with 100 ml water; 30 ml of this decoction is to be given orally three times daily before meals.	Kidney and bladder	Decoction (oral)	0.32
21.	*Matricaria aurea* (Loefl.) Sch.Bip./ Golden Chamomile/Babonaj thahabi/ Pharm-PCT-1519	*Compositae*	Flowers/ In case of lung cancer: Boil 50 grams flowers with 500 ml water; the vapor is inhaled twice daily for 10 minutes each time. In case of liver and prostate cancers: Boil 30 grams from the flowers with 300 ml water for 15 minutes; 100 ml from the decoction is to be given orally twice daily.	Lung, liver and prostate	Vapor inhalation	0.47
22.	*Onopordum cynarocephalum* subsp./Artichoke Cotton-thistle/ kondrees/ Pharm-PCT-1692	*Compositae*	Flowers/ Steep 50 grams of the dried flowers with 100 ml water for one night; 50 ml from this infusion is to be given three times daily.	Stomach and colon	Infusion (oral)	0.23
23.	*Silybum marianum* (L.) Gaertn./Milk thistle Khorfeesh/Pharm-PCT-2282	*Compositae*	Stalk/ 30 drops of celery stalk fresh juice is to be given orally every three hours.	Colon and skin cancer	Fresh juice (oral)	0.32
24.	*Taraxacum syriacum Boiss.*/ Common dandelion/Hindeba/ Pharm-PCT-2396	*Compositae*	Leaves/ Boil about 60 grams from the dried powdered plant with 100 ml water for 15 minutes; entire decoction is to be given 3–5 times daily.	Pancreatic and gallbladder stomach	Decoction (oral)	0.35
25.	*Citrullus colocynthis* (L.) Schrad./ Bitter Gourd/Hanthal/Pharm-PCT-628	*Cucurbitaceae*	Fruits/ About 150 grams from the powdered dried fruits mixed with equal quantity of lanolin; the produced cream applied topically twice daily.	Skin	Cream (topical)	0.13
26.	*Cucumis sativus* L./cucumber/ kheyar/ Pharm-PCT-2737	*Cucurbitaceae*	Seeds/ Steep 4grams of the ground seeds with 100 ml water for 12 hours; 10 ml from this infusion is to be given three times daily	Colon	Infusion (oral)	0.07
27.	*Ecballium elaterium* (L.) A.Rich./ Exploding cucumber / Ketha’ alhemar/ Pharm-PCT-870	*Cucurbitaceae*	Fruits (juice)/ / One fresh fruit pulp (about 25gram) juice is to be orally given five times daily	Throat and liver	Fresh juice (oral)	0.17
28.	*Ephedra alata* Decne./ Ephedra/ Alanda/ Pharm-PCT-904	*Ephedraceae*	Entire plant/ About 100 grams of the powdered plant boiled with 500 ml water for 5 minutes; 100 ml of this decoction is to be given orally twice a day.	Brain, liver and colon	Decoction (oral)	0.72
29.	*Arbutus andrachne* L./Greek Strawberry Tree/ Kotlob/ Pharm-PCT-213	*Ericaceae*	Fruits/ Boil 50 grams of the ground fruits with 100 ml water and 100 gram sugar; 20 ml of the produced syrup is to be given orally 5–6 times daily.	Stomach	Syrup (oral)	0.06
30.	*Euphorbia hierosolymitana* Boiss./ Spurge/Halablabon/Pharm-PCT-988	*Euphorbiaceae*	Entire plant/ Boil 50 grams from the plant with 150 ml water for 10 minutes; 5 ml of this decoction is to be given each 8 hours.	Ovarian, breast and prostate	Decoction (oral)	0.59
31.	*Quercus calliprinos* Webb/ Palestine oak/ Baloot/ Pharm-PCT-1978	*Fagaceae*	Fruits/ Boil about 20 grams from the fruits with 100 ml water, 20 ml of this decoction is to be given four times daily.	Colorectal	Decoction (oral)	0.11
32.	*Quercus ithaburensis* Decne./ Valonia oak/Sendnyan/ Pharm-PCT-1980	*Fagaceae*	Bark/ Mix 30 grams of the powdered bark with 100 ml lanolin then apply this cream on the cancer area.	Skin	Cream (topical)	0.13
33.	*Hypericum perforatum* L./St.John’s Wort/ Oshbat ala’ran/Pharm-PCT-2734	*Hypericaceae*	Flowers/ About 100 grams of the powdered flowers with 100 ml olive oil then filtered and 20 ml of the produced infusion is to be given orally twice daily.	Brain	Infused in olive oil (oral)	0.05
34.	*Crocus sativus* L./ Saffron/ Za’faran/ Pharm-PCT-2733	*Iridaceae*	Flowers/ Two grams of Saffron powder steeped in 300 ml camel milk; this milk infusion is to be given early morning once daily.	Liver and kidney	Infusion (oral)	0.46
35.	*Melissa officinalis* L./Balm mint/ Torenjan/ Pharm-PCT-1564	*Lamiaceae*	Aerial parts/ About 10 grams of the fresh leaves are given orally five times daily.	Lung and non-Hodgkin lymphoma	Fresh plant (oral)	0.27
36.	*Origanum jordanicum* Danin & Kunne/Thyme/Za'atar/Pharm-PCT-1729	*Lamiaceae*	Leaves/ About 50 grams of the leaves boiled in 500 ml water; inhale vapor three times daily, five minutes each time.	Lung, throat cancer	Vapor inhalation	0.15
37.	*Rosmarinus officinalis* L./ Rosemary/ Hasa alban/ Pharm-PCT-2732	*Lamiaceae*	Leaves/ About 20 grams of the leaves boiled in 600 ml water; inhale vapor three times daily, about ten minutes each time.	Lung cancer	Vapor inhalation	0.10
38.	*Salvia fruticosa* Mill./ Sage/ Maryamya/ Pharm-PCT-2117	*Lamiaceae*	Aerial parts/ Boil about 70 grams from the leaves with 300 ml water, the decoction is to be given four times daily.	Colon and liver	Decoction (oral)	0.26
39.	*Salvia palaestina* Benth./Kosa’en(kharna) falestini/ Pharm-PCT-2124	*Lamiaceae*	Leaves/ Fifteen grams of the leaves steeped with 100 ml water for 12 hours; 10 ml from this infusion is to be given twice a day.	Brain	Infusion (oral)	0.19
40.	*Teucrium capitatum* L./ Teucrium/ Ja’da/ Pharm-PCT-2407	*Lamiaceae*	Entire plant/ About 150 grams of the plant boiled for 10 minutes with water; 30 ml from the produced decoction is to be given once daily.	Pancreatic and liver	Decoction (oral)	0.42
41.	*Laurus nobilis* L. / Bay/ Gaar/ Pharm-PCT-1366	*Lauraceae*	Leaves/ Ten grams from the dried leaves boiled with 100 ml water; 20 ml of this decoction is to be given before meals 3–4 times daily	Prostate	Decoction (oral)	0.25
42.	*Alhagi graecorum* Boiss./ Camel-thorn/ ala’alook/ Pharm-PCT-65	*Leguminosae*	Fruits/ About 50 grams from the dried fruits boiled in 300 ml water; 10 ml of this decoction is to be given orally twice daily.	Glandular	Decoction (oral)	0.13
43.	*Glycine soja* Siebold & Zucc./ Soy/ Soya/ Pharm-PCT-2731	*Leguminosae*	Seeds/ About 100 grams of the seeds boiled in 500 ml water; 100 ml of this decoction is to be given orally 5–7 times daily.	Breast, ovarian and Hodgkin lymphoma	Decoction (oral)	0.19
44.	*Ononis viscosa* subsp. sicula (Guss.) Hub.-Mor./ spiny restharrow/ Shabrak(wassem)/ Pharm-PCT-1686	*Leguminosae*	Entire plant/ About 20–30 grams from the powdered plant boiled in 350 ml water; 10 ml of this decoction is to be given twice daily.	Prostate, stomach and breast	Decoction (oral)	0.07
45.	*Linum usitatissimum* L./ Flax/ Ketan/ Pharm-PCT-2735	*Linaceae*	Seeds/ Ground Seed (10 grams) are to be given orally three times daily.	Ovarian, breast and colon	Powder (oral)	0.19
46.	*Lawsonia inermis* L./ Henna / Hena/ Pharm-PCT-2736	*Lythraceae*	Leaves/ A paste from crushed fresh leaves (about 30 grams) are applied externally to affected areas.	Skin	Paste (topical)	0.22
47.	*Punica granatum* L./ Pomegranate/ Romman/ Pharm-PCT-2730	*Lythraceae*	Fruits (peels)/ About 500 grams of the fruit peels boiled in 1 liter water with 1000 grams sugar; 50 ml of this syrup is to be given twice daily.	Colorectal	Syrup (oral)	0.27
48.	*Ficus sycomorus* L./ Sycamore Fig / Jomeez/ Pharm-PCT-1030	*Moraceae*	Fruits/ One fresh fruit is boiled with 100 ml syrup for five minute; 20 ml of the syrup is to be given orally 4–6 times daily.	Lung	Syrup (oral)	0.19
49.	*Psidium guajava* L./ Guava /Juafa/ Pharm-PCT-2720	*Myrtaceae*	Leaves/ A decoction of 100 grams leaves is prepared in one liter water; 2–3 cups are taken orally per day until improvement occurs.	Lung and stomach	Decoction (oral)	0.19
50.	*Orobanche aegyptiaca* Pers. / Broomrape/ Halook/ Pharm-PCT-1746	*Orobanchaceae*	Roots/ About 500 grams of the ground roots boiled in one liter water for 30 minutes,; 50 ml of this decoction is to be given orally once daily.	Ovarian and breast	Decoction (oral)	0.21
51.	*Trifolium philistaeum* var. filifolium Zohary/ Palestine Clover/ Barsem/ Pharm-PCT-2493	*Papilionaceae*	Flowers/ About 50 grams of the flowers boiled in 60 ml water; 30 ml of this decoction is to be taken internally twice a day.	Ovarian, breast and non Hodgkin lymphoma	Decoction (oral)	0.14
52.	*Plantago lanceolata* L./ narrowleaf plantain/ Lesan alhamal/ Pharm-PCT-1887	*Plantaginaceae*	Leaves/ About 500 grams of the leaves boiled in 500 ml water; 100 ml of this decoction is to be taken internally 3–5 times a day.	Throat	Decoction (oral)	0.29
53.	*Triticum aestivum* L./ Bread wheat/ Kameh/ Pharm-PCT-2540	*Poaceae*	Seeds (husk)/ Powdered seed husks (10 grams) are given orally three times daily.	Colon	Powder (oral)	0.44
54.	*Portulaca oleracea* L./ Little Hogweed/ Farfahena/ Pharm-PCT-1935	*Portulacaceae*	Aerial parts/ About 100 grams of the plant boiled in 500 ml water; 30 ml of this decoction is to be taken 3–5 times a day.	Stomach and esophageal	Decoction (oral)	0.47
55.	*Cyclamen persicum* Mill./ Cyclamen/ Sapoon alraa’e/ Pharm-PCT-777	*Primulaceae*	Roots/ Twenty five grams of the ground roots boiled in 350 ml water; 30 ml of this decoction is to be given orally twice daily.	Prostate and bladder	Decoction (oral)	0.21
56.	*Nigella arvensis* L./ Black cumin / Kezha/ Pharm-PCT-1640	*Ranunculaceae*	Seeds/ About 100 grams of the ground seeds boiled in 330 ml water for 10–15 minutes; this decoction is to be taken 4–5 times daily	Lung, brain and skin	Decoction (oral)	0.49
57.	*Ziziphus spina-christi* (L.)Desf./ Christ's Thorn Jujube/ Cedar/ Pharm-PCT-2693	*Rhamnaceae*	Flowers/ About 100 grams of the flowers boiled in 500 ml water; 50 ml of this decoction is to be given internally 4–7 times daily.	Lung	Decoction (oral)	0.31
58.	*Crataegus azarolus* L./ Azarole Hawthorn/ Za’ror/ Pharm-PCT-712	*Rosaceae*	Fruits/ One kilogram of fresh fruit boiled with 1000 ml syrup for 30 minute; 50 ml of the syrup is to be given 5–6 times per day.	Lung	Syrup (oral)	0.41
59.	*Galium aparine* L./ Stickyweed/ Satoor/ Pharm-PCT-1069	*Rubiaceae*	Leaves/ Fifty grams of the leaves steeped with 100 ml water for one night; 10 ml from this infusion is to be given twice daily.	Hodgkin Lymphoma	Infusion (oral)	0.11
60.	*Salix alba* L./ White Salix/ Sofsaf abyad/ Pharm-PCT-2093	*Salicaceae*	Bark/ About 60 grams from the plant boiled with 500 ml water for 10 minutes; 50 ml of this decoction is to be given orally each 6 hours.	Colon	Decoction (oral)	0.19
61.	*Viscum cruciatum* Sieber ex Boiss./ Mistletoe/ hedal/ Pharm-PCT-2662	*Santalaceae*	Leaves/ About 500 grams from the powdered plant boiled with one liter water for 10 minutes; 50 drops of this decoction is to be given three times daily.	Esophageal	Decoction (oral)	0.51
62.	*Acer obtusifolium* Sm./ Syrian Maple/ Kaikab/ Pharm-PCT-15	*Sapindaceae*	Fruits/ About 500 grams of the fresh fruits boiled with 100 ml water and 100 gram sugar; 20 ml of the resulting syrup is to be given 6–8 times per day.	Throat and lung	Syrup (oral)	0.21
63.	*Verbascum sinuatum* L./ Mullein/ A’awarwar/ Pharm-PCT-2604	*Scrophulariaceae*	Leaves/ Five grams of the leaves boiled with 150 ml water for 30 minutes; 15 ml of this decoction is to be given orally twice daily.	Breast	Decoction (oral)	0.59
64.	*Capsicum annuum* L/ Chili pepper/ Shatta/ Pharm-PCT-2729	*Solanaceae*	Fruits/ Four fruits (100 grams) from the plant boiled with 200 ml water for 20 minutes; 5 drops of this decoction is to be given orally each 8 hours.	Skin, bladder	Decoction (oral)	0.16
65.	*Lycium europaeum* L./ Box thorn/ A’wsaj/ Pharm-PCT-1487	*Solanaceae*	Fruit/ About half kilogram from the fruit boiled with 500 ml water for one hour; 50 ml of this decoction is to be given once daily.	Bladder, prostate and breast	Decoction (oral)	0.37
66.	*Mandragora autumnalis* Mill./Mandrake/ Tofah almajan/ Pharm-PCT-1509	*Solanaceae*	Fruits/ Ten grams from the plant boiled with100 ml water for 30 minutes, 5 drops of this decoction is to be given twice daily.	Lung	Decoction (oral)	0.21
67.	*Withania somnifera* (L.) Dunal/ Ashwagandha/ A’eba’b monawem/ Pharm-PCT-2678	*Solanaceae*	Roots/ About 50 grams of the ground roots steeped with 100 ml water for 24 hours; 10 ml from this infusion is to be given twice daily.	Esophageal, skin and prostate	Infusion (oral)	0.19
68.	*Camellia sinensis* (L.) Kuntze /Green Tea/ Shae akhdar/ Pharm-PCT-2706	*Theaceae*	Leaves/ About 50 grams from the plant boiled with 300 ml water for 10 minutes; this decoction is to be given 6–8 times daily.	Breast, lung and ovarian	Decoction (oral)	0.31
69.	*Daphne linearifolia* L./ Mezereon/ Mazeryon/ Pharm-PCT-825	*Thymelaeaceae*	Fruits/ A decoction is prepared from 7 to 8 fruits (about 100 grams) boiled in 1 liter water and taken orally, 1 ml two times per day and taken for 14 days.	Lung	Decoction (oral)	0.04
70.	*Urtica urens* L./ Small Nettle/ Korees harek/ Pharm-PCT-2562	*Urticaceae*	Aerial parts/ About 100–120 grams of the plant boiled with 500 ml water for 30 minutes; 50 ml of the decoction is to be given three times daily.	Bones and stomach	Decoction (oral)	0.51
71.	*Curcuma longa* L./ Turmeric/ Korkom/ Pharm-PCT-2709	*Zingiberaceae*	Rhizomes/ About 500 grams from the ground dry rhizomes boiled with one liter water for 30 minutes; 300 ml of this decoction is to be given orally three times daily.	Prostate, bladder and liver	Decoction (oral)	0.53
72.	*Zingiber officinale* Roscoe/ Ginger/ Zangabil/ Pharm-PCT-2724	*Zingiberaceae*	Rhizomes/ About 100 grams of the ground dry rhizomes are boiled in 300 ml water for 10 minutes and given twice daily after meals.	Stomach and liver	Infusion (oral)	0.51

### Data analysis

All citations were placed into ailment categories for each type of cancer. Factor of informant’s consensus (F_ic_) was employed to indicate how homogenous the information is. In fact, its main use is to select disease categories where there is consensus on the use of plants among the informants. F_ic_ value is close to 0 if plants are chosen randomly, or if informants do not exchange information about their use. High values of F_ic_ (close to 1) occur when there is a well-defined selection criterion in the community and/or if information is frequently exchanged between informants [28].

The F_ic_ is calculated as in the following equation:

$$ {F}_{ic}=\frac{Nur-Nt}{Nur-1} $$

Where Nur is the number of use citations in each category and Nt is the number of taxa used.

Fidelity level (Fl) was defined as the ratio between the number of informants who independently suggested the use of a species for the same major purpose and the total number of informants who mentioned the plant for any use. Fl is of equal importance to F_ic_ and it can be calculated according to the following equation*:*

$$ Fl=\frac{Np}{N}*100 $$

Where Np is the number of informants that reported a use of a plant species to treat a particular disease and N is the number of informants that used the plants as a medicine to treat any given disease [29].

The use-value (UV) is a quantitative method that can be used in order to prove the relative importance of species known locally. It is can be calculated according to the following equation:

$$ \mathrm{U}\mathrm{V}-\frac{{\displaystyle \sum U}}{n} $$

Where UV is the use value of a species; *U* the number of citations per species; *n* the number of informants [30].

Results of calculated F_IC_, Fl and UV are shown in Tables 2, 3 and 4.

**Table 3:** Factor of informant’s consensus (F_ic_) categorized by the types of cancer

	Types of cancer	Nt	Nur	F_ic_
1	Lung	15	350	0.96
2	Colorectal	14	345	0.96
3	Liver	13	364	0.97
4	Skin	13	564	0.98
5	Stomach	11	265	0.96
6	Breast	11	267	0.96
7	Prostate	9	172	0.95
8	Bladder	8	159	0.96
9	Ovarian	6	84	0.94
10	Brain	5	122	0.97
11	Throat	4	74	0.96
12	Kidney	4	96	0.97
13	Esophageal	4	157	0.98
14	Bone	3	194	0.99
15	Hodgkin's lymphoma	2	19	0.94
16	Non-Hodgkin's lymphoma	2	25	0.96
17	Pancreatic	2	57	0.98
18	Spinal cord	1	25	1
19	Gallbladder	1	24	1

**Table 4:** Fidelity level of herbal medicines mentioned

Medicinal plant	Type of cancer	Np	N	FL,%
*Achillea aleppica* DC.	Colon	13	13	100.00
*Alhagi graecorum* Boiss.	Skin	88	88	100.00
*Arbutus andrachne* L.	Skin	3	3	100.00
*Brassica oleracea* L.	Skin	91	91	100.00
*Calotropis procera* (Aiton) Dryand.	Skin	70	70	100.00
*Capparis spinosa* L.	Skin	79	79	100.00
*Catharanthus roseus* (L.) G.Don	Liver	69	69	100.00
*Citrullus colocynthis* (L.) Schrad.	Bone	71	71	100.00
*Colchicum hierosolymitanum* L.	Bones cancer	71	71	100.00
*Crataegus azarolus* L.	Skin	79	79	100.00
*Cucumis sativus* L.	Liver	16	16	100.00
*Daphne linearifolia* L.	Skin	19	19	100.00
*Daucus guttatus* Sm.	Colon	10	10	100.00
*Ficus sycomorus* L.	Stomach	9	9	100.00
*Galium aparine* L.	Colorectal	17	17	100.00
*Hypericum perforatum* L.	Skin	20	20	100.00
*Laurus nobilis* L.	Brain	8	8	100.00
*Lawsonia inermis* L.	Lung	15	15	100.00
*Mandragora officinalis* Mill.	Brain	28	28	100.00
*Mangifera indica* L.	Prostate	37	37	100.00
*Nerium oleander* L.	Glandular	19	19	100.00
*Pistacia palaestina* Boiss.	Skin	33	33	100.00
*Plantago lanceolata* L.	Colorectal	40	40	100.00
*Punica granatum* L.	Lung	29	29	100.00
*Quercus calliprinos* Webb	Throat	43	43	100.00
*Quercus ithaburensis* Decne.	Colon	66	66	100.00
*Rosmarinus officinalis* L.	Lung	47	47	100.00
*Salix alba* L.	Lung	61	61	100.00
*Salvia palaestina* Benth.	Hodgkin's Lymphoma	16	16	100.00
*Sinapis arvensis* L.	Colon	29	29	100.00
*Triticum aestivum* L.	Esophageal	77	77	100.00
*Verbascum sinuatum* L.	Breast	89	89	100.00
*Viscum cruciatum* Sieber ex Boiss.	Lung	31	31	100.00
*Ziziphus spina-christi* (L.)Desf.	Lung	6	6	100.00
*Origanum jordanicum* Danin & Kunne	Lung	18	23	78.26
*Psidium guajava* L.	Lung	21	29	72.41
*Orobanche aegyptiaca* Pers.	Breast	23	32	71.88
*Urtica urens* L.	Bones	52	77	67.53
*Zingiber officinale* Roscoe	Stomach	52	77	67.53
*Arum dioscoridis* Sm.	Stomach	99	99	66.67
*Allium cepa* L.	Stomach	29	44	65.91
*Teucrium capitatum* L.	Pancreatic	41	63	65.08
*Portulaca oleracea* L.	Esophageal	42	71	59.15
*Salvia fruticosa* Mill.	Colon	23	39	58.97
*Melissa officinalis* L.	Non-Hodgkin's lymphoma	24	41	58.54
*Inula viscosa* (L.) Aiton	Kidney	28	48	58.33
*Crocus sativus* L.	Kidney	39	69	56.52
*Acer obtusifolium* Sm.	Lung	18	32	56.25
*Onopordum cynarocephalum* subsp.	Colon	19	34	55.88
*Petroselinum crispum* (Mill.) Fuss	Kidney	13	24	54.17
*Capsicum annuum* L	Bladder	13	24	54.17
*Trifolium philistaeum* var. filifolium Zohary	Ovarian	11	21	52.38
*Annona muricata* L.	Bladder	25	48	52.08
*Ecballium elaterium* (L.) A.Rich.	Liver	13	25	52.00
*Glycine soja* Siebold & Zucc.	Ovarian	15	29	51.72
*Cyclamen persicum* Mill.	Bladder	16	31	51.61
*Cichorium endivia* L.	Colon	17	33	51.52
*Curcuma longa* L.	Bladder	41	80	51.25

## Results and discussion

Traditional herbal medicine knowledge and their utilization by indigenous cultures are not only useful for conservation of biodiversity and cultural traditions but also useful for the population’s healthcare and drug discovery in the present and in the future [31, 32].

Several studies have shown that around 80 % of rural populations in the developing countries consider herbal remedies as integral parts of treatments available. Recently, the use of natural herbal products is increasing in both developed and developing countries due to many reasons [33, 34].

As shown in (Table 1), most of the respondents who work in this field were males. Most of them had educational level equal or higher than high school. In fact about 19.3 % of the total interviewed were university graduates. The table also showed that the majority of respondents were from areas of the West Bank that mostly depend on agriculture or grazing as a mean of income (Jenin, Nablus, Salfeit and Toulkarem). In fact these areas are geographically close to each other (Fig. 1).

Notably the results of this research have revealed that 72 plant species belonging to 44 families were frequently used for treatment of cancer by the 150 herbalists, traditional practitioner healers, rural dwellers and people of the West Bank, Palestine (Table 2).

**Fig. 2:**
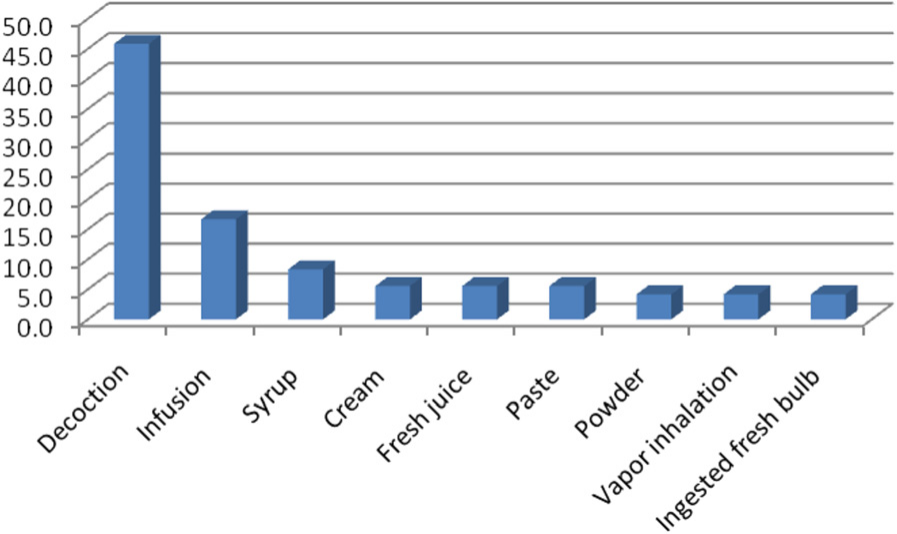
Frequency of methods of preparation from herbals

Echoing our findings in this research, members of the family *Compositae* and *Lamiaceae* were the most commonly used as presented in Table 2. The methods of preparation were decoctions (boiling the plants parts in water), infusions (steeping the plants in water for limited time), syrup (boiling the plants with water and sugar (1:1), creams (mixing the plant powders with goat fat or lanolin), paste, fresh juice, ingested entire plant, powder and vapor inhalations. Decoctions and infusions were the most frequently used methods of preparation as presented in Fig. 2.

**Fig. 3:**
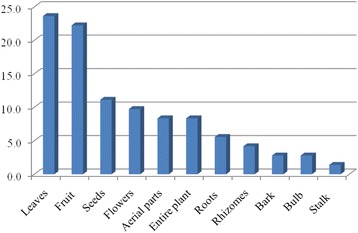
Frequency of the used parts of medicinal plants in cancer treatment

Leaves, fruits and seeds were reported to be the most frequently used parts of plants for the treatment of cancer, constituting about 56.9 % of the preparations. This was followed by flowers, aerial parts, entire plants, roots, rhizomes, barks, bulb and stalks as presented in Fig. 3.

**Fig. 4:**
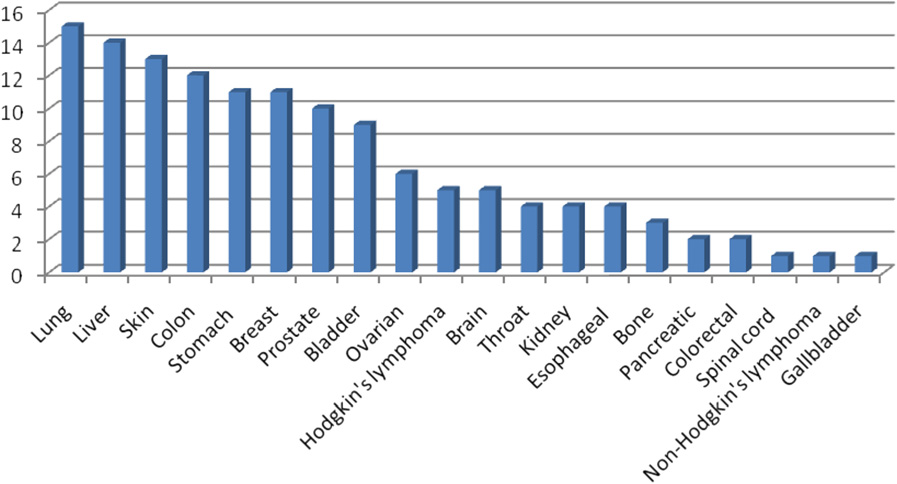
Cancer types treated with herbals in Palestine

The most common cancer type treated with herbal remedies was lung cancer followed by liver, skin, colon and breast cancers as reported in (Fig. 4).

This research shows that the medicinal plants still play a role in the care of cancer patients in Palestine. *Ephedra alata*, *Arum dioscoridis*, *Arum palaestinum* were the most commonly used medicinal plants for treatment of cancer and all of these three plants were prepared as decoctions.

However, *Ephedra alata* were reported to treat three different types of cancer (brain, liver and colon). Accordingly, three Fl values were calculated. The highest one (40.74 %) was for liver cancer. The same consideration can be raised for *Arum palaestinum* which was reported to treat four different cancers (Liver, colon, kidney and breast). Breast cancer showed the highest Fl value (45.74 %). According to table 4, only Fl values higher than 50 % were included.

In the Mediterranean region and especially in the Holy land (Palestine), the traditional medicine has been highly appreciated and trusted. Many patients go to herbalists or informants to get benefit from this field. Most practitioners are males and this was confirmed in this study; some of them have university degrees. Complementary and alternative medicines are widely used among cancer patients throughout the world. In a previous study from Palestine, 60.9 % of cancer patients reported using medicinal herbs [35]. Medicinal plants utilized in indigenous health traditional system are gradually becoming wiped out due to over utilization, human overpopulation and from other human impact on the environment. The main problem is destructive harvesting of the subterranean parts of the medicinal plants, or even the entire plant.

Medicinal plants maintain the health and vitality of individuals, and may help in treatment of various diseases, including cancer. In this study, some anticancer medicinal plants of foreign origin have been presented. Many of these medicinal plants possess good immunomodulatory and antioxidant properties, which may lead to anticancer activities. The antioxidant phytochemicals protect the cells from oxidative damage. Thus, consuming a diet rich in antioxidant plant foods (e.g. fruits and vegetables) will provide health-protective effects. In 2013, a traditional practitioner living in Jenin claimed that he could cure a cancer patient completely using *Ephedra alata*. The local media here were interested in this story and many researchers have started working on this plant.

A multidisciplinary approach combining traditional herbal knowledge with pharmaceutical research is a valuable method for identifying potential herbs with possible clinical significance in cancer care [36].

To achieve a positive response to herbal preparations, the proper part of the plant that contains the active constituents should be chosen. It is well known that not all the plant parts contain the same concentration of the active constituents. The other factors to be considered are the harvesting time of the herb (collection time), the soil, the climate conditions, and the method of drying, processing, and extraction [37, 38]. Methods of preparation were mainly decoction and infusion. This is similar to previous studies in our country conducted by Ali-Shtayeh et al., 2011 and Jaradat, 2005 [35, 39]. In an ethnopharmacological survey of medicinal herbs in Golan Heights and the West Bank region performed by Said et al., 2002, only seven plant species were found to treat cancer among 81 species used for treating 115 different ailments and diseases [40]. In another study conducted in the West Bank, Ali-Shtayeh and Rana, 2011, found only 25 plant species used for treatment in cancer without mentioning which type of cancer they can treat [41]. In another study conducted by Ali-Shtayeh et al., 2011, in Palestine, 58 plant species were collected for cancer treatments [35], while in the survey which was conducted by Hudaib et al., 2008 in Jordan, Mujib Nature Reserve and surrounding area, only six plant species were found for treatment of cancer also without mentioning the type of cancer [42].

The methods of preparation mentioned by the informants are not supported by scientific evidence; this could be suitable for some plants but not for the others. In fact, the boiling process can cause severe degradation of the medicinal components in some plants.

The dosage is another concern. To have the expected benefits, the patients should receive a fixed well defined dosage, but in traditional medicine the suitable doses are not clear [37], so studies are needed to determine the concentration of active ingredients depending on their method of preparation to give the suitable recommended doses.

Table 3 shows the F_ic_ values calculated for the categorized cancers. F_ic_ values obtained for the reported cancers indicate the degree of shared knowledge among informants for the treatment of a cancer by certain medicinal plants. Most cancers had high F_ic_ values; however the highest F_ic_ =1 was scored for spinal cord and gallbladder cancers. Fl was 100 % for many plants; the highest UV (0.72) was for *Ephedra alata*.

Most of the mentioned plants in Table 2 are edible plants and the most of the non-edible plants were used externally. Therefore minor restrictions could be included as they have minor risk compared to the obtained therapeutic benefits from their use for cancer.

Resulting from this information, scientific practical work is in progress on the phytochemical and pharmacological analysis of the plants. This study is important to preserve the knowledge of medicinal plants used by the people of the West Bank regions. Also, it is of significance to utilize new therapeutic natural plant products in various treatments of diseases. Moreover, clinical studies approved according to international guidelines should be considered in order to prove the safety and efficacy of treatment, especially for the most frequently reported medicinal plants.

## Conclusion

Many plant species are still used by herbalists and traditional practitioner healers in Palestine for treating various types of cancer. This article provides the knowledge about anticancer medicinal plants of local and foreign origin, which are used by the people all over the Palestinian area in addition to their method of preparation. Many of the used plants have been approved scientifically to have some anticancer activity. A combined scientific effort between informants and the scientific community working in this field may help in the discovery of new anticancer agents. Moreover, scientists may explore the most convenient method of extraction, formulation and dose determination in order to achieve the best benefit from these plants.

Further scientific laboratory studies are required to explore and to investigate the safety and efficacy of these plants, their potential therapeutic effects as well as probable interactions of these medicinal products with conventional anticancer medicines.
